# Community awareness and experiences of health workers concerning mosquito-borne viral diseases in selected districts of Gambella Region, Southwestern Ethiopia

**DOI:** 10.1080/20008686.2021.1988453

**Published:** 2021-11-01

**Authors:** Getahun Asebe, Gezahegne Mamo, Barbara Wieland, Girmay Medhin, Getachew Tilahun, Woldaregay Erku Abegaz, Mengistu Legesse

**Affiliations:** a Addis Ababa University College of Veterinary Medicine, Department of Veterinary Microbiology, Immunology and Public Health, Bishoftu, Ethiopia; b College of Agriculture and Natural Resources, Gambella University, Gambella, Ethiopia; c International Livestock Research Institute, Addis Ababa, Ethiopia; d Aklilu Lemma Institute of Pathobiology, Addis Ababa University, Addis Ababa, Ethiopia; e College of Health Sciences, School of Medicine, Department of Microbiology, Immunology & Parasitology, Addis Ababa University, Addis Ababa, Ethiopia

**Keywords:** Qualitative, arboviruses, community participation, a mosquito-transmitted disease, Ethiopia

## Abstract

In this study, we assessed community awareness and experiences of health workers about mosquito-borne viral diseases in selected districts of the Gambella Region, South Western Ethiopia. A community and health facility-based qualitative study involving 11 focus group discussions (FGDs) with community dmembers and two FGDs with health workers was conducted between November 2017 to January 2018. A total of 122 community members and 16 health workers participated in the study. All the discussants mentioned malaria, typhoid fever, unknown causes of diarrhea and skin diseases as the major public health problems in the area. Using pictures of *Anopheles* and *Aedes* mosquitoes, participants confirmed that both mosquitoes are present in the area. They identified *Anopheles* as the vector of malaria. However, community discussants could not mention the name of a disease that can be transmitted by *Aedes* mosquito though *they* mentioned that Aedes mosquito bites both humans and animals during the day time in forest areas and causes skin itching to humans. Meanwhile, community participants from Pakag, a village bordering South Sudan, expressed concern that *Aedes* mosquito can cause a malaria-like disease which can kill within a few days. Health workers from Itang health center described that in 2016, an outbreak of an unknown disease that causes fever and jaundice occurred and killed seven individuals in a village called Akula, which is closer to a South Sudan refugee camp. Overall, the findings showed that community members and health workers in the area do not have adequate information on mosquito-borne viral diseases. Creating awareness, improving laboratory services and further epidemiological studies would be important for early warning and preparedness for outbreaks in the area.

## Introduction

In many low- and middle-income countries (LMIC), reports on emerging and re-emerging mosquito-borne viral diseases caused by yellow fever virus (YFV), dengue virus (DENV), West Nile virus (WNV), chikungunya virus (CHIKV), Rift Valley fever virus (RVFV), and Zika virus (ZIKV) are becoming more frequent [1–3]. Among other things, yellow fever (YF) remains a major public health problem in many African countries despite the availability of effective vaccines [4–7].

Since the largest outbreak ever recorded in East Africa in 1960–1962, which caused 100,000 cases and 30,000 deaths, YF outbreaks of various magnitude have occurred repeatedly in Ethiopia [8]. In 1966, a YF outbreak caused 2,200 cases and 450 deaths in Ethiopia [9] and between November 2012 and October 2013 YF re-appeared in the South Omo Zone of Southern part of the country and resulted in 43 deaths [10]. In recent years, outbreaks of dengue fever (DF) have also occurred in the Eastern part of Ethiopia [11,12]. Viruses such as Zika, West Nile, Chikungunya, Wesselsbron, Talaguine, and Sindbis were reported in the Gamo Gofa and Wollega areas [13]. In the case of RVF, there were no reports of active cases in Ethiopia in both human and animals, but one IgM positive animal was detected from Somali and Borena areas [13]. In Ethiopian situation, a reliable study has not been done so far for many arboviruses except conducting survey during outbreak conditions. On the other hand, studies on *Aedes* and *Culex* mosquitoes have been done in many geographical areas of Ethiopia [14,15].

Similar to many African countries, in Ethiopia, factors like weak surveillance and case finding systems, poor health infrastructures, and shortage of diagnostic facilities, a close interaction of humans and reservoir animals, wide distribution of the mosquito vectors in the country as well as low level of community knowledge of mosquitoes-borne viral diseases are contributing to re-emergence and also hamper the control of many arboviruses [16,17]. A successful strategy in the prevention and control of arboviral diseases depends on many factors, for example, giving health education for the communities, training of health workers as well as the building of health infrastructures for regular surveillance and diagnosis of viruses [18].

Most environments in tropical and subtropical countries are suitable for the breeding of mosquitoes that transmit mosquito-borne viral diseases [19–21]. Nevertheless, there is little information on the knowledge of communities [16,22] and experiences of health workers about mosquito-borne viral diseases in high-risk areas of Africa [23].

Despite the proximity of the Gambella Region to the Southern Nation and Nationalities Peoples Region of Ethiopia, where the YF outbreak was recently reported [10], and it being closer to South Sudan where many arbovirus cases occurring frequently [24–26], and the high risk of arboviruses being introduced because of migration of refugees, animals, and wildlife across the border between South Sudan and the Gambella Region, so far there was no such kind of study focused on the assessment of risk indicators such as the presence of potential vectors, previous incidence history, and reservoir hosts for the outbreak of mosquito-borne viral diseases. In this study, we assessed community awareness and health workers’ experiences about mosquito-borne viral diseases as well as knowledge of mosquito-vectors in selected districts of the Gambella Region, Southwestern Ethiopia.

## Methods

### Study area and population

Gambella Region is one of the nine regions of the Federal States of Ethiopia and geographically located in the Southwestern part of the country between latitudes 6° 22ʹ and 8° 30ʹ N and longitudes 33° 10ʹ and 35° 50ʹ E, bordering South Sudan internationally. The Region has about 436, 000 population [27]. The Region administratively divided into three zones (Nuer, Anuak, and Mejeng), 13 districts (one special district and 12 districts), and 247 kebeles (smaller administrative units) where the districts are part of the zones and kebeles are under the districts. The study was conducted in two districts, namely, Itang special district and Lare district. The districts were purposively selected because of their proximity to South Sudan, where YF and RVF outbreaks were recently reported [25,26]. Moreover, the districts host many refugees and migratory pastoralists from South Sudan. Hence, we purposefully included five ‘kebeles’ (the lowest administrative structure in the district) from Itang special district and four ‘kebeles’ from Lare by considering the proximity of the “kebeles’ to the refugee camps and border of South Sudan.

### Study design and data collection

Between November 2017 and January 2018, a community and health facility-based qualitative study was conducted in the selected kebeles of the two districts. In the selected kebeles, a total of 11 FGDs (five with women and six with men, consisting of 8–12 participants per FGD) were conducted. In the FGDs, individuals aged over 18 years were enrolled, and discussions with men and women were made separately at respected sites. The participants of the FGDs were recruited with the help of the administrator/chairperson of the respective kebeles.

**Figure 1.**
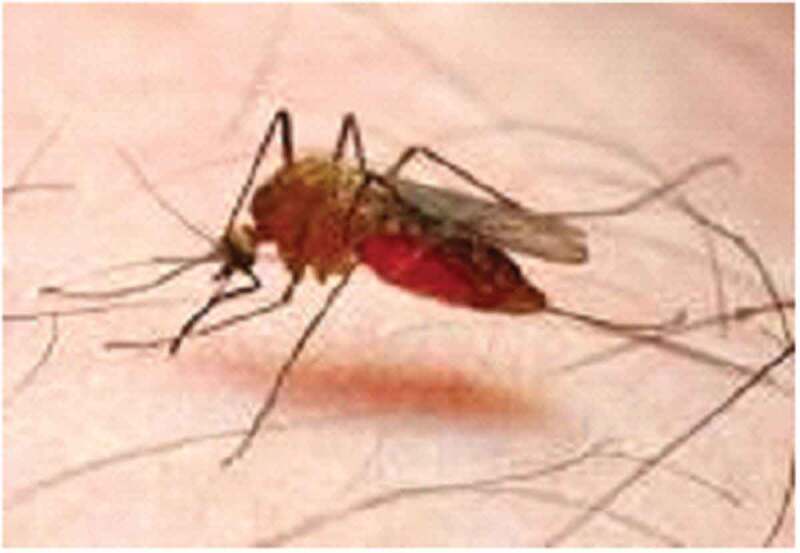
(a) Picture of Anopheles mosquito [29]

To ensure consistency in data collection across FGDs, a checklist consisting of points to be discussed was prepared. The contents of the checklist include mentioning of major public health problems in the area, sources of the diseases, the presence of *Anopheles* and *Aedes* mosquitoes (supported with pictures Figure 1a & b) [28,29]. The mosquitoes were discussed at genera level (where species level of discussion not known in the area), biting time and place, type of diseases that these mosquitoes transmit, whether they have seen/encountered YF-like disease (supported with a picture of a patient with yellowing of the eyes due to YF and other explanations by showing different body parts like head for headache, bleeding eye, nose and mouth, back pain, and muscle for muscle pain for other mosquito-borne viral diseases), and prevention of mosquitoes biting.

**Figure:**
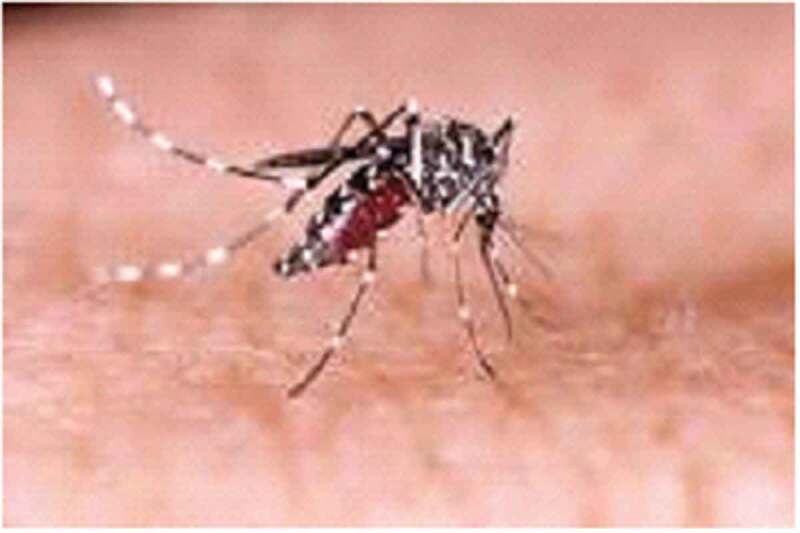

One FGD was conducted in each district with health workers that focused on details of major health problems encountered in the area, common mosquito-borne viral diseases such as YF, DF, RVF, and the like, diagnosis and treatment of febrile patients and their recommendations on the management of unknown febrile cases.

The health centers were selected based on their location and health workers were also selected purposely based on their service years, duration of stay in the study areas and their qualification. The FGD discussion was moderated by the research team and trained health workers and recorded using a voice recorder as well as notes. The discussion session was administered using a checklist containing various topics and mediated through a translator who is trained and who can communicate the respective local languages and the Amharic language for better exploring of the targeted issues. The facilitator speaks in Amharic, while the translator mediates in both the local languages (Anuak and Nuer) and commence an immediate translation for the note-making person and audio recording device simultaneously. The checklists were prepared in both Amharic and English with an equivalent meaning.

### Data management and analysis

All the information gathered from FGDs was transcribed verbatim and translated from local languages (Nuer and Anuak) into the national language (Amharic) and then, into English. The data were analyzed using content analysis of the FGD discussant responses in each group.

## Results

### Socio-demographic characteristics of the study participants

At the community level, a total of 122 participants (60 men and 62 women, age range 19–73 years, median 43.5 years) participated in the FGDs. Most of the study participants (94.6%) were pastoralists and had no education (84.4%) (Table 1). A total of 16 health workers (age range 24–36 years, median 30 years) participated in the FGD at health centers (Table 2).

**Table 1.** Sociodemographic characteristics of participants involved in the community-based FGD

Variables	Number of participants	Percent (%)
**District**		
*Itang*	56	45.9
*Lare*	66	4.1
**Gender**		
Men	60	49.2
Women	62	50.8
**Age category**		
19–24 years	5	4.09
25–44 years	56	45.9
45–64 years	55	45.08
≥65 years	6	4.9
**Education**		
Illiterate	103	84.4
Diploma	1	0.8
Secondary school	18	14.8
**Occupation**		
Agro pastoralist	7	5.7
Pastoralist	115	94.3
**Marital status**		
Married	120	98.4
Others	2	(1.6
**Ethnic group**		
Nuer	118	96.7
Anuak	4	3.3

**Table 2.** Socio-demographic characteristics of health workers

Variables	Number of participants	Percent (%)
**Health center**		
*Itang*	6	37.5
*Lare*	10	62.5
**Qualification of health workers**		
Clinical nurses	12	75.0
Health Officer	2	12.5
Laboratory technician	2	12.5
**Age category**		
19–24 years	1	6.2
25–44 years	15	37.5
**Work experience in the area**		
2–5 years	9	56.3
Above 5 years	7	43.8

### Community knowledge about diseases of public health importance

Male participants listed diseases such as malaria, typhoid, tuberculosis (TB), hemorrhoid, HIV/AIDS, and diseases caused by herpes zoster virus infection (locally known as disease caused by spider urine) as common public health problems in the areas. Some study participants mentioned clinical signs/symptoms rather than mentioning the disease by name, and the public health concerns were expressed as clinical syndromes or signs/symptoms such as fever, diarrhea, headache, stomach ache, back and joint pain, sudden death, vomiting, coughing, and skin diseases.

Besides, men participants from one of the study sites (Nip Nip kebele) mentioned that unknown diseases characterized by high fever, joint and back pains can be transmitted from animals to humans through the consumption of milk from sick animals or consumption of meat of dead animals. The participants also complained about diseases caused by drinking and/or using pond water for cooking food.

Similarly, women participants have mentioned diseases such as malaria, typhoid, and TB as the major public health problems of the areas. They also outlined disease signs/symptoms rather than their names such as skin diseases, diarrhea, joint problems, disease caused by spider urine and fever-related diseases.

One woman said ‘*There is a disease that we do not know its name or its cause. Its signs/symptoms are bloody diarrhea, fever, and joint pain. It affects all age groups and both sexes’*.

### *Community knowledge about diseases transmitted by* Anopheles *and* Aedes *mosquitoes*

When shown pictures of *Anopheles* mosquitoes, almost all the male and female participants identified *Anopheles* mosquitoes (locally known as Nyise in Nuer language and Bewo in Anuak language) as the vector of malaria. They also mentioned that malaria is common during the rainy season and the vector bites both during the day in forest areas and also during the night at home. The participants identified malaria by its clinical signs/symptoms such as fever, headache, back and/or joint pain, and weakness. They also mentioned that using bed nets and clothes like blanket can protect against mosquito bites and prevent malaria.

However, there is a misperception among women participants as some of believed that rain is also the cause of malaria ‘when a person is showered with rain, or stayed outside in the rain, he/she will develop malaria’. One woman also mentioned that malaria can be transmitted from mother to child through breastfeeding.

All women and men recognized *Aedes* mosquitoes (locally known as forest black-and-white color Nyise in Nuer and Bewo in Anuak). They also indicated that *Aedes* mosquitoes bite both humans and animals during the day time in the forest areas. However, the participants expressed that they had no information on the specific disease that can be transmitted by *Aedes* mosquitoes. They said that biting by *Aedes* mostly results in itching. Very few of the participants mentioned *Aedes* can cause malaria and some other unknown diseases. One old man said ‘*We know another disease that causes bleeding through nose and mouth. It seems malaria, but it is fatal and some individuals may die within a week and others stay for a while*’. Men and women participants from Pakag kebele (a village on the border of South Sudan) Lare district strongly argued that biting by this *Aedes* mosquito can cause malaria-like diseases which can kill within a few days due to bleeding through the nose/mouth and/or by changing eye color to yellowish. They added that the disease has occurred every year since 1991 during the rainy season or after the rainy season when the population of mosquitoes is abundant. They said that the disease killed greater than 10 individuals in the area between August and the beginning of September 2017. They thought that the disease came from other places like South and North Sudan because of migration of pastoralists and refugees.

One woman from Itang special district said ‘*Some years ago I have seen a person who was sick and his eyes became yellowish’. The woman added, ‘that disease is characterized by signs/symptoms, like fever, bleeding through nose and mouth and it is a killer disease which has no treatment’. Another woman from Lare district said, ‘a year ago my six years old son was sick from a disease which caused eyes, palm and fingers to develop a yellowish color, but he recovered without any treatment within about seven days’. She added, ‘I also remember a 12-years old boy from a neighbor who was also sick from a similar disease, but at that time it was said to be malaria and he recovered*’.

### Health workers experience about major public health problems in the area

Health professionals from both Itang and Lare health centers mentioned diseases such as malaria, diarrhea, sexually transmitted diseases, pneumonia, typhoid, malnutrition, TB, HIV, and visceral leishmaniasis as major health problems. However, almost all the participants said that they did not encounter or had no information on any disease other than malaria that can be transmitted through mosquito bites in the area. Sometimes, they may run out of Rapid Diagnostic Test (RDT) or reagents used for malaria diagnosis, and they treat a patient for malaria based on clinical examinations. If treated individuals might not recover from their illness, then health workers suspect typhoid fever and administer anti-typhoid drugs. If a person treated for malaria and typhoid still does not get relief from his/her illness, the health workers refer the patient to the Regional hospital.

When health workers were asked whether they had encountered YF or other mosquito-borne viral disease cases in the area and how they diagnose them, they said they had not encountered YF or other mosquito-borne viral disease cases in the area. However, during the discussion, health workers from the Itang health center mentioned that they suspected YF – like cases, which caused the death of seven individuals from 10 suspected cases in 2016 in a village called Akula that is close to a South Sudan refugee camp. They also stated that they do not have the facility to diagnose and rule out cases of YF or other mosquito–borne viral diseases among febrile patients who found negative for malaria or typhoid fever.

## Discussion

The results of this study revealed a lack of community awareness about different mosquito-borne viral diseases in general, although the study area can be considered a high-risk site. However, the study participants had a high level of knowledge about malaria. There are many possible explanations for the lack of community awareness about mosquito-borne viral diseases. For instance, in areas like Gambella, where malaria is highly endemic, it should not be surprising to find low awareness of people on mosquito-borne viral diseases since community members mainly focus on malaria, while other mosquito-borne viral diseases remain unrecognized. Besides, in resource-limited countries, there is a high chance of misdiagnosis of mosquito-borne viral infections due to the lack of routine diagnostic tests and the non-specific nature of clinical symptoms of febrile causing diseases [30].

A previous study on community awareness about YF in Southern Ethiopia indicated that community members had a great difficulty in identifying YF from malaria (especially from falciparum malaria) [17] which is similar with the findings of this study. Another study in Tanzania also revealed that many community members believed that most instances of fever are due to malaria and the community had a low level of awareness about other non-malaria febrile illnesses like RVF or DF despite the endemicity of these diseases [23]. On the other hand, very few studies have shown relatively good community awareness of arbovirus diseases that differ from the findings of the current study [31].

In the present study area, mosquitoes are locally known as ‘Nyise’ in Nuer and ‘Bewo’ Anuak language. Almost all the participants recognized the *Anopheles* mosquito as the one that transmits malaria. They also correctly mentioned its breeding sites, biting time and prevention using bed nets. However, some individuals argued that *Anopheles* mosquito can bite both during night and day time in a dark place, or inside a house. Thus, the findings of the present study showed better knowledge on the *Anopheles* mosquito compared to a study conducted in Jamaica [32] that could be due to the endemicity of malaria in the present study area. Moreover, communities’ knowledge towards the prevention of *Anopheles* mosquitoes would play a big role in the tackling of malaria.

The discussants are also familiar with the *Aedes* mosquito, as they have identified it as black-and-white color forest Nyise in Nuer and Bewo in Anuak language. They also know that Aedes mosquito breeds inside waterbodies in a forest area during the rainy season. Participants from different kebeles also underlined that the *Aedes* mosquito bites both humans and their animals during the day time in forest areas or near waterbodies, but it does not come to their homes like that of *Anopheles*. A community-based study in rural Cambodia also revealed a high level of knowledge regarding *Aedes* breeding and biting time, similar to the findings of our study [33].

However, the majority of the participants (except participants from Pakag village) expressed that they do not know what kind of disease *Aedes* mosquito transmits following biting other than causing an intermittent irritating and itching of skin, which implies that the community members in the present study area had no information regarding the role of *Aedes* mosquito in the transmission of arboviruses. A previous study in Jamaica showed very poor community knowledge of a disease that can be transmitted through the biting of *Aedes* mosquito [32]. In a study conducted in Kongwa and Kilombero districts in Tanzania, a very small percentage of participating community members were aware of vectors of RVF suggesting the difficulty about prevention of RVF [22]. Different studies also showed poor communities’ knowledge about the role of *Aedes* mosquito as a vector of different arboviral diseases such as DF and YF [17,34].

Health workers from Itang health center expressed their concerns about the risk of occurrence of YF in the area, because of the frequent migration of refugees/pastoralists from YF endemic countries, primarily from South Sudan and Kenya to Gambella. The proximity of Gambella Region to countries like South Sudan, where repeated mosquito-borne viral disease outbreaks have been reported [25], and the high risk of mosquito-borne viruses to be introduced due to migration of refugees, animals, and wildlife across the border, as well as the ecological suitability of the region for mosquito vectors would highly contribute to the occurrence and transmission of mosquito-borne viruses in the area.

Some study participants’ responses from the Lare (Pakag village) and Itang districts suggested the occurrence and transmission of mosquito-borne viral diseases in the area. Community members mentioned some clinical signs of mosquito-borne viral disease despite the fact that the signs and symptoms are more or less similar to each other and counted as malaria cases.

Taken together, all the evidences collected in this study would imply the occurrence and transmission of mosquito-borne viral diseases in the present study area, which would not be surprising given the high risk for these diseases to occur due to the frequent arboviral diseases report in the neighboring countries together with the regular and irregular free movement of animals and people across the borders. Unexpected outbreaks of major mosquito-borne viral diseases such as YF, DF, and RVF have been becoming major public and animal health problems in East African countries since the early 1950s [8, 10, 35, 36; 12, 26, 37]. However, in many cases, infection with mosquito-borne viruses causes subclinical or clinical signs/symptoms that are confused with other diseases such as malaria [4,30] which was also reflected during the current focus group discussion and warrants strengthening of surveillance and increasing community awareness.

In the present study area, the diagnosis of febrile cases, in general, is rarely supported or confirmed by laboratory tests, reflecting the limitations of the health service delivery system and the shortage of laboratory facilities during the study period. This would likely lead to under diagnosis and reporting of the actual presence of mosquito-borne viral diseases in the area. Upon health workers’ discussion, the poorly equipped laboratories in the Region are not able to provide simple and urgently needed routine disease diagnosis; and even ruling out typhoid fever is a big challenge in the area. This kind of problem is also common in other African countries [23,30,38].

This study would provide important information on community awareness and health workers’ experiences about major mosquito-borne viral diseases in the study area. However, the study was conducted in purposely selected districts of the Gambella Region, and the findings cannot be generalized to all the districts of the Region. In addition, this qualitative study was not supported by quantitative data, which would be important to provide additional detailed information on community knowledge about mosquito-borne viral diseases in the area.

## Conclusion

The present study showed that community members and health workers in the study areas do not have adequate information on mosquito-borne viral diseases despite the presence of potential mosquito vectors. However, evidence collected in this study suggests the occurrence and transmission of mosquito-borne viral diseases in the present study area. Hence, there is a need to strengthen population-based surveillance of mosquito-borne viral diseases and potential vectors, taking preventive measures, creating awareness among health-care providers and community members. It is also important to appreciate the need to provide diagnostic testing for the health institutions of the Region to ensure early detection and preparedness for these public health threats.

## Data Availability

The datasets used and/or analyzed during the current study are available from the corresponding author on reasonable request.

## Abbreviations

CHICK: chikungunya fever; DF: dengue fever; FGDs: focus group discussions; IRB: Institutional Review Board; LMIC: low- and middle-income countries; RDT: Rapid Diagnostic Test; RVF: Rift Valley Fever; WNV: West Nile Virus; YF: yellow fever; Zv: Zika virus.
